# Microbiota-bone axis in ageing-related bone diseases

**DOI:** 10.3389/fendo.2024.1414350

**Published:** 2024-07-15

**Authors:** Liangxuan Fu, Peilin Zhang, Yicheng Wang, Xiaonan Liu

**Affiliations:** Department of Orthopedics, Shanghai Sixth People’s Hospital Affiliated to Shanghai Jiao Tong University School of Medicine, Shanghai, China

**Keywords:** aging, skeletal degenerative diseases, bone metabolism, gut microbiota, bone

## Abstract

Bone homeostasis in physiology depends on the balance between bone formation and resorption, and in pathology, this homeostasis is susceptible to disruption by different influences, especially under ageing condition. Gut microbiota has been recognized as a crucial factor in regulating host health. Numerous studies have demonstrated a significant association between gut microbiota and bone metabolism through host-microbiota crosstalk, and gut microbiota is even an important factor in the pathogenesis of bone metabolism-related diseases that cannot be ignored. This review explores the interplay between gut microbiota and bone metabolism, focusing on the roles of gut microbiota in bone ageing and aging-related bone diseases, including osteoporosis, fragility fracture repair, osteoarthritis, and spinal degeneration from different perspectives. The impact of gut microbiota on bone metabolism during aging through modification of endocrinology system, immune system and gut microbiota metabolites are summarized, facilitating a better grasp of the pathogenesis of aging-related bone metabolic diseases. This review offers innovative insights into targeting the gut microbiota for the treatment of bone ageing-related diseases as a clinical therapeutic strategy.

## 1 Introduction

Bone is an organ that experiences continuous remodeling by osteoblasts and osteoclasts. Osteoblasts are responsible for the production of type I collagen, osteocalcin, and alkaline phosphatase, which serve as scaffolds for the deposition of calcium and phosphorus, facilitating the formation of new bone (1). On the contrary, osteoclasts, responsible for bone resorption, attach to the bone surface and secrete hydrogen ions, leading to the dissolution of the bone matrix and the release of mineral deposits from the bone. During aging, debilitating skeletal diseases like osteoporosis occur due to the inability to maintain bone homeostasis. This phenomenon primarily arises from the overstimulation of osteoclasts or the suppression of osteoblasts, leading to a higher rate of bone resorption compared to bone formation.

Somatic cells have a limited lifespan. During the 1960s, Leonard Hayflick first described the limited replicative potential of normal cultured human fibroblasts and termed this phenotype “cellular senescence” (2). These cells are causally implicated in aging and in an ever-expanding list of diseases (3). Accumulating evidence has indicated that aging is often accompanied by degradation of the bone microenvironment, leading to decreased differentiation and compromised bone repair capacity (4). As the human body undergoes the ageing process, senescent cells occur and release senescence-associated secretory phenotype (SASP) factors, including IL-6 and GCA, into the bone microenvironment (5). The escalating burden of senescent cells with age disrupts tissue structure and function, emerging as a significant factor in elevating disease susceptibility and mortality among the elderly. Osteoporosis, sarcopenia, degenerative disc disease, and osteoarthritis are musculoskeletal disorders that are closely related to the ageing process. Focus on more detailed aspects. The aging behavior of stem cells, macrophages and osteocytes plays an important role in the aging of bone tissue (6). It has been demonstrated that senescence of skeletal stem cells in mice leads to greater release of pro-inflammatory and pro-resorptive factors into the bone senescence microenvironment leading to bone senescence (7). Senescent macrophages have also been shown to influence bone senescence by promoting inflammation and the release of granular calprotectin (GCA) (8). Also, chronic inflammation accelerates the aging of immune cells, leading to weakened immune function and an inability to remove senescent cells and inflammatory factors, resulting in a vicious cycle of inflammation and aging. As for the osteocytes, previous studies have suggested that possible characteristics of aged osteocytes include, but are not limited to, impaired mechanosensitivity, accumulated cellular senescence, dysfunctional perilacunar/canalicular remodeling (PLR), and degenerated lacuna-canalicular network (LCN). The aging osteocytes can exhibit mechanical- and endocrine-responsive properties to the aging microenvironment (9). Targeted elimination of senescent cells from ageing tissues has the potential to postpone the onset and progression of age-related diseases. Additionally, counteracting oxidative and inflammatory stressors may decelerate the formation of senescent cells, thereby offering insights into potential therapeutic interventions (10).

**Figure 1:**
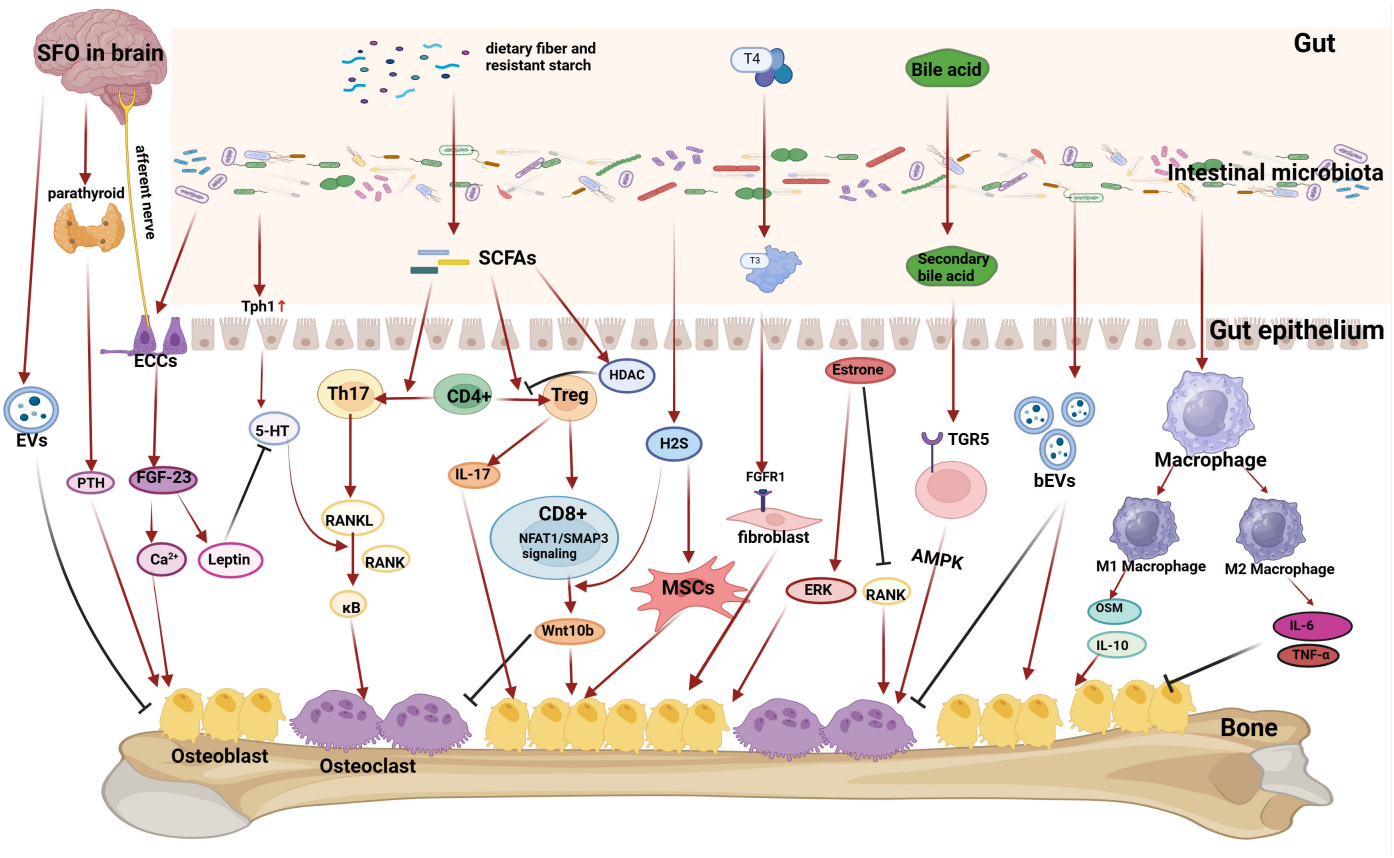
Mechanisms by which gut microbiota affect bone metabolism. (1) Gut microbiota can act on intestinal secretory cells (ECCs), which can affect the subpial region of the brain (SFO) through the gut-brain axis, and then affect the release of parathyroid hormone from the parathyroid glands, which ultimately affects the absorption of calcium and bone formation, and also affect the serum calcium level and thus affect the formation of bone by influencing the FDF-23 content, and also affects the regulation of the leptin content and the role of 5-HT. The brain can also release extracellular vesicles (EVs) to inhibit osteoblasts after stimulation of the gut-brain axis. (2) Gut microbiota can affect 5-HT absorption by upregulating Tph1 protein expression in intestinal epithelial cells, and 5-HT can amplify the role of the RANKL pathway in promoting osteoclast differentiation. (3) Gut microbiota can break down dietary fiber and other substances to produce short-chain fatty acids (SCFAs), which regulate the Th17-Treg balance, and then play a dual role in regulating osteoblasts and osteoclasts differentiation through the RANKL pathway or the Wnt10b pathway. Gut microbiota can break down food to produce H2S, which acts on mesenchymal stem cells (MSCs) to promote osteoblast differentiation. ( 4) The gut microbiota can influence material transformation to regulate bone metabolism. Gut microbiota plays a crucial role in the conversion of thyroid hormone T4 to a more active form of T3, which can act on the FGFR1 receptor of fibroblasts to promote osteoblast differentiation. Gut microbiota plays an important role in the conversion of bile acids into secondary bile acids, which can act on the TGR5 receptor of cells to activate the AMPK pathway to promote osteoblast differentiation. (5) Gut microbiota can influence the amount of estrogen. Increasing estrogen levels in postmenopausal women by influencing gut microbiota can promote osteogenesis and improve osteoporosis. Gut microbiota can also release bacterial-derived extracellular vesicles (bEVs) that act on bone to influence bone metabolism. (6) Gut microbiota can affect macrophage differentiation. M2-type macrophages can secrete factors such as OSM and IL-10 to promote osteogenesis, while M1-type macrophages can secrete pro-inflammatory factors such as TNF-α and IL-6 to inhibit osteogenesis and promote osteoblastogenesis.

The interactions between gut metabolism and bone health have been investigated, encompassing various mechanisms such as gut metabolites, the immune system, intestinal epithelial barrier function, nutrient absorption metabolism, endocrinology, hormone function, and modulation of the gut-brain-skeleton axis to impact bone metabolism (**Figure 1**). It is well known that gut microbes occupy a central position in gut metabolism. Intestinal microorganisms encompass a diverse array of commensal and pathogenic microorganisms, such as bacteria, archaea, viruses, and fungi, that colonize the intestinal mucosa. Comprising 1014 species of bacteria and possessing 150 times more genes than the human body, this entity has been referred to as the “forgotten organ of the human body” (11)*. Firmicutes* and *Bacteroidetes* constitute the main components of the gut microbiota in both mice and humans (12). The ratio of the two is of great importance for bone metabolism and the improving of the ratio is advantageous to suppress age-induced chronic inflammation and oxidative damage in skeletal tissue (13).

The gut microbiota represents a crucial element of the gut barrier (14), the integrity of the gut barrier is closely associated with gut permeability. Dysbiosis of the gut microbiota may contribute to the development of systemic low-grade inflammation and the activation of the immune system by causing intestinal barrier dysfunction, increased intestinal permeability and leakage of toxic bacterial metabolites into the circulation (15). And numerous studies have indicated that this progress are linked to a multitude of processes that govern bone mass, mechanical function, marrow production, development, metabolism, osteoporosis (16), inflammation, fracture risk, and cancer in bone.

**Figure 2:**
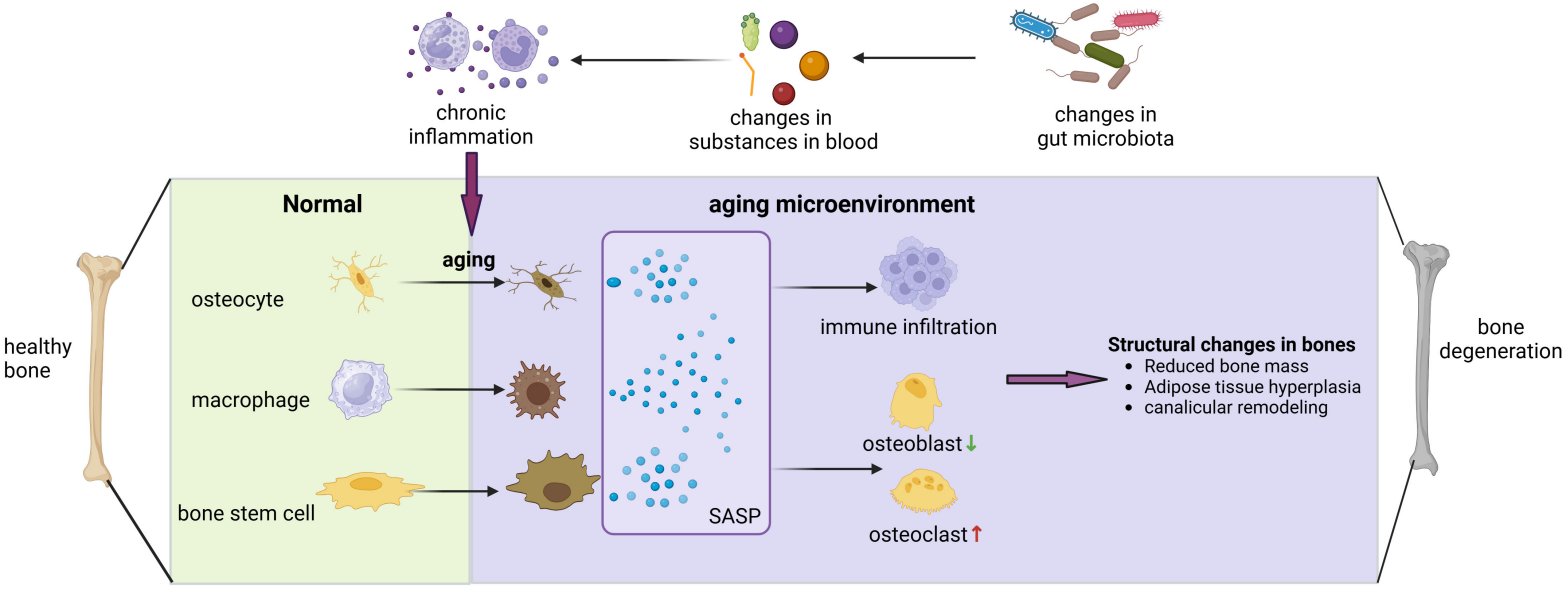
Gut microbiota can contribute to bone degenerative diseases by affecting the bone aging microenvironment. Alterations in the composition of gut microbiota can affect LPS entry, bile acid metabolism, short-chain fatty acid absorption, and other processes that induce widespread chronic inflammation in the body, and chronic inflammation has been shown to have a close association with cellular senescence. When osteoblasts, macrophages, and skeletal stem cells become senescent, they release SASP, which exacerbates the inflammatory infiltration of the bone microenvironment, promotes osteoblasts and inhibits osteogenesis, leading to various changes in bone structure and ultimately triggering the development of degenerative bone diseases.

Throughout the process of co-evolution with the host, the composition of the gut microbiota undergoes continual modifications as individuals age, transitioning from infancy to old age. It is inevitable that changes in the gut microbiota occur concomitantly with organismal ageing. The gut microbiota composition in older adults is distinguished by diminished diversity and stability, decreased expression of genes responsible for producing short-chain fatty acids (SCFA), reduced capacity to break down glycoconjugates, heightened proteolytic activity, and, at the genus level, an increase in Proteobacteria enrichment. The enrichment of Proteobacteria, along with an elevated proportion of the Bacteroidetes and Clostridium genera, was observed (17). Various metabolites present in the gut microbiota exhibit potent anti-inflammatory and antioxidant properties, potentially playing a role in mitigating the development of inflammatory and tumorigenic conditions linked to the ageing process. Dysbiosis of the gut can lead to chronic inflammatory stress in the body, potentially contributing to cellular senescence and worsening degenerative bone diseases (18) (**Figure 2**).

## 2 Mechanism of gut microbiota metabolites affecting bone metabolism

### 2.1 Metabolites of gut microbiota

The gut microbiota significantly impacts bone metabolism through its modulation of short-chain fatty acids (SCFAs). SCFAs are primarily generated through the fermentation of carbohydrates like dietary fiber, resistant starch, and oligosaccharides by anaerobic bacteria in the colon. SCFAs can inhibit LPS-induced inflammatory responses, and the uptake of sufficient SCAFs may be associated with the inhibition of senescence. It has been demonstrated that SCFAs can bind to GPR41 (FFAR3)/GPR43 (FFAR2) receptors on cell membranes or passively diffuse into cells to activate histone deacetylase (HDAC) to block the NF-κB signaling pathway and inhibit the inflammatory response (19). SCFAs also directly exhibit a dual regulatory effect on osteoclasts. SCFAs have the capacity to promote the differentiation of regulatory T cells (Treg) through the inhibition of histone deacetylase, thus SCFAs can attenuate osteoclast differentiation and formation. Conversely, SCFAs can also prompt naive T cells to differentiate into T helper 17 (Th17) cells, which secrete the receptor activator of nuclear factor-κB ligand (RANKL). This, in turn, triggers the activation of nuclear factor-κB upon binding to RANK. The nuclear factor-κB promotes the expression of osteoclasts. SCFAs have the capacity to influence osteoblasts by prompting naïve CD4+ cells to transform into Treg cells within the bone marrow. They also facilitate the interaction between NFAT and SMAD, leading to the activation of Wnt10b and subsequently triggering Wnt signaling in osteoblasts. This process ultimately enhances bone formation (20). Additionally, SCAFs can enhance bone formation through the regulation of IGF-1 production. SCAFs can also activate osteoblasts by inducing the expression of glucagon-like peptide-1 (GLP-1), which plays a crucial role in osteoblast function. The secretion of glucagon-like peptide-1 (GLP-1) aims to improve bone strength (21).

The gut microbiota can also act on bone via 5-HT. Intestinal-derived 5-HT, which accounts for 95% of 5-HT *in vivo*, is mainly synthesized by enterochromaffin cells via the rate-limiting enzyme [tryptophan hydroxylase 1 (Tph1)], and it has been reported that bacteria of the genus Clostridium stimulate the production of 5-HT by enteroendocrine cells by inducing the expression of Tph1 in enterochromaffin cells (22). Cui et al. reported that inhibition of Tph1 expression affected osteoblast proliferation and differentiation causing high bone mass syndrome (23). Stone et al. reported that intestinal-derived 5-HT amplified the effects of RANKL on osteoclastogenesis in mice (24). Thus, The gut microbiota plays a crucial role in modulating the interaction between osteoblasts and osteoclasts through its impact on the synthesis of the pivotal rate-limiting enzyme, Tph1, thereby influencing the levels of enteric 5-HT.

Additionally, the gut microbiota can modulate bone metabolism through bile acids. Bile acids are substances that facilitate digestion. Upon entering the intestine, primary bile acids undergo conversion into secondary bile acids through the influence of the gut microbiota. The membrane-bound G protein-coupled bile acid receptor (TGR5) serves as the receptor for secondary bile acids. The interaction between secondary bile acids and TGR5 has the potential to stimulate osteoclast differentiation via the AMPK signaling pathway. The differentiation of osteoclasts can be influenced by the gut microbiota, thereby regulating bone metabolism through the modulation of bile acid conversion (25). The relationship between bile acids and aging has also been demonstrated. Studies have shown that when bile acid metabolism is disturbed after adverse changes in the structure of the gut microbiota there is a significant increase in the body’s inflammation levels and an increase in the incidence of aging-related diseases, including Alzheimer’s disease and cardiovascular disease (8), but the relationship between bile acids and bone aging diseases remains to be demonstrated.

### 2.2 Affecting substance absorption

The gut microbiota can impact bone metabolism through its influence on nutrient absorption and modulation of nutrient levels within the body. On the one hand, The production of short-chain fatty acids (SCFAs) leads to a direct decrease in the pH of the intestinal lumen (26), enhancing mineral solubilization which leads to the conversion of more calcium into a soluble form. This soluble calcium then permeates intestinal cells via passive exchange, thereby augmenting the body’s calcium absorption. Consequently, this process influences bone formation. On the other hand, A fraction of the substance undergoes degradation within the gastrointestinal tract, specifically in the intestines, leading to the generation of bioactive peptides that exhibit various biological activities. Small peptide molecules have the ability to adsorb, bind, and transport calcium and other essential elements within the human body, thereby enhancing their absorption and utilization rates. For instance, casein phosphopeptide, which acts as a carrier for Ca2+, has been documented to exhibit preventive effects against osteoporosis in ovariectomized mice (27). Wang et al. discovered that the gut microbiota has the ability to reprogram lipid absorption and metabolism in the mouse intestine through the inhibition of long-chain non-coding RNA (lncRNA) Snhg9 expression in small intestinal epithelial cells (28).

### 2.3 Modulating the immune system

As described in the previous section on the senescence microenvironment, when senescent cells are present in the bone metabolic environment and secrete SASP or when the level of inflammation is increased due to the regulation of other factors it can induce bone degenerative diseases by promoting immune responses, such as elevating the level of inflammatory factors such as IL-6, recruiting neutrophils, and inducing the conversion of macrophages to the M1 type to disrupt the equilibrium between osteoclasts and osteoblasts (5). Gut microbiota can play an important role in the inflammatory level of the bone metabolic environment by, for example, releasing LPS into the bloodstream to induce an immune response, and thus the gut microbiota-immune system-bone senescence microenvironment is an important pathway for the disruption of bone homeostasis and the development of disease.

Previous studies have suggested the function of T cells in affecting bone metabolism. TGF-β and IL-2 stimulate T cells to differentiate into Treg cells. Additionally, TGF collaborates with certain pro-inflammatory factors like IL-6 and IL-21 to enhance the differentiation of naïve T cells into Th17 cells (29). On one hand, regulatory T (Treg) cells possess the capability to impede osteoclast maturation and differentiation via a pathway mediated by cytotoxic T lymphocyte-associated protein 4 (CTLA-4) (30). On the other hand, Treg cells can regulate the production of Wnt10b in CD8+ T cells (31), which in turn can trigger the activation of the Wnt/β-catenin signaling pathway. The reduction of beta-catenin levels is linked to diminished bone formation. This dynamic is evident in the Th17-Treg homeostasis, where a higher differentiation of CD4+ T cells into Treg cells leads to the inhibition of osteoclasts and a facilitation of bone formation.

Macrophages also play an important regulatory role in bone metabolism and bone aging. F4/80 macrophages located in proximity to the periosteal and endosteal surfaces, commonly referred to as “osteomacs,” have been demonstrated to oversee the mineralization process of osteoblasts in laboratory settings and uphold the functionality of mature osteoblasts in living organisms (*in vivo*) *(* 32). Furthermore, oncostatin M (OSM) and interleukin-10 (IL-10) secreted by macrophages have been shown to promote the differentiation and mineralization of osteoblasts (5). The inhibition of osteogenesis is caused by TNF-α and IL-6, which are produced by activated M1-like macrophages (33). In the context of immune-mediated bone metabolism, the RANKL (receptor activator of NF-kappa B ligand)-RANK-OPG axis and the immunoreceptor tyrosine-based activation motif (ITAM) pathway are pivotal in regulating both normal bone remodeling and pathological bone conditions (34).

The gut microbiota has the capacity to regulate bone metabolism through its impact on the immune system. In terms of affecting the T cells, the *probiotic bacterium Lactobacillus acidophilus* has been shown to inhibit bone loss in mice that have undergone ovariectomy (OVX) by modulating the balance between regulatory T cells (Treg) and T helper 17 cells (Th17) (35). The colonization of gnotobiotic mice with Indigenous *Clostridium species* led to a heightened accumulation of colonic Tregs, consequently fostering bone formation (36). T regulatory (Treg) cells have the capacity to secrete IL-17, which is crucial for bone formation *in vivo* and has been shown to alleviate osteoporosis in mice post-ovariectomy (OVX). Additionally, filamentous bacteria have been found to enhance the production of IL-17, suggesting a potential contributory role in bone development (37). In terms of affecting Macrophage, it is reported that the gut microbiota can promote Bone Marrow-Derived Macrophages to secret pro-inflammatory factors like IL-6, IL-12p40 (38). And IL-6, IL-12/p40 have been reported to be closely associated with bone ageing diseases such as osteoarthritis and osteoporosis (39).

### 2.4 Affecting the internal secretion

From a local perspective of gastrointestinal hormones, the gastrointestinal (GI) tract is recognized as one of the largest endocrine organs in the human body. Enteroendocrine cells (ECCs) in GI play a crucial role in secreting a diverse array of gastrointestinal hormones that exert a significant regulatory influence on bone metabolism. For instance, leptin is synthesized by primary and P cells in the gastric fundus region. It has been documented to potentially stimulate fibroblast growth factor 23 (FGF-23) (40), influencing osteocalcin, 5-HT, serotonin levels, and other pathways that modulate bone growth (41). Glucagon-like peptide-1 (GLP-1) has been documented to stimulate bone formation and suppress bone resorption (42). Meanwhile, the use of a GLP-1 receptor agonist activated the PKA/CREB pathway to exhibit anti-inflammatory activity in a rat model of osteoarthritis, suggesting that GLP-1 may be associated with the formation of the skeletal senescence microenvironment and influence the onset of degenerative bone diseases (43). The gut microbiota has the capacity to impact the secretion of gastrointestinal hormones by intestinal secretory cells, thereby potentially influencing bone metabolism (44).

From a systemic perspective of the endocrine system, the gut microbiota can concurrently impact the activity of various hormones, including thyroid hormones, sex hormones, growth hormones, and other hormones, thereby exerting an indirect influence on bone metabolism. The gut microbiota plays a significant role in the conversion of thyroid hormones. Approximately 20% of thyroxine (T4) is transformed into the active form of triiodothyronine (T3) within the intestine. T3, in turn, stimulates the proliferation and differentiation of osteoblasts by activating Fibroblast Growth Factor Receptor 1 (FGFR1) (45). The presence of excessive pathogens or inflammation in the intestinal tract can reduce the conversion of thyroid hormones in the gut. Consequently, bone reconstruction is impacted, potentially resulting in osteoporosis. Estrogen deficiency has been associated with bone loss (46). In a study where estrogen-deficient mice were administered Lactobacillus acidophilus, a reduction in bone resorption markers and an improvement in bone formation were observed (47). The colonization of germ-free mice with gut microorganisms has been shown to markedly elevate serum insulin-like growth factor-1 levels, thereby stimulating bone growth (48).

### 2.5 Through gut-brain-bone axis

The gut-brain axis refers to a complex network of bidirectional connections that integrate neural, hormonal, and immune signals between the gastrointestinal tract and the brain. On one hand, signals originating from the brain impact gastrointestinal homeostasis processes through the autonomic nervous system and the hypothalamic-pituitary-adrenal axis. On the contrary, the gut microbiota can also be detected through numerous metabolites by specialized cells in the gut, such as enteroendocrine cells (EEC), enterochromaffin cells (ECC), and primary or secondary afferent nerve endings. These cells are responsible for perceiving bacterial metabolites and subsequently transmitting neural signals to the brain. The gut microbiota has the capacity to engage with immune cells within the gut, resulting in both local and systemic immune activation. This interaction leads to the generation of circulating metabolites that can traverse the blood-brain barrier, reaching the central nervous system and exerting a direct influence on neural activity. Consequently, this process impacts bone metabolism through the intricate mechanism known as the brain-bone axis.

With regard to the brain-bone axis, Yang et al. found that activation of GABAergic neurons and Glutinergic neurons in specific subfornical organ (SFO) regions of the brain using chemical genetics resulted in decreased or increased PTH hormones, respectively, which in turn resulted in decreased or increased bone density (49). Shen et al. found that brain-derived extracellular vesicles(EVs) in Alzheimer’s disease(AD) patients significantly inhibited osteogenic differentiation of BMSCs and promoted their lipogenic differentiation, inducing bone loss and fat accumulation in the bone marrow cavity (50). Given that gut microbiota has many effects on the brain, and that the brain also plays an important role in the regulation of bone metabolism, the gut-brain-bone axis will be a very promising area of study.

### 2.6 Via extracellular vesicles

Bacterial extracellular vesicles (bEVs) are functional nanovesicles secreted by a variety of gut microbiota bacteria. These vesicles contain a diverse array of biomolecules derived from the parent bacteria and play a role in numerous signaling pathways within the host organism. BEVs are linked to systemic diseases, such as bone disorders. Xie et al. introduced a new model for regulating bone metabolism through Enterobacteriaceae functional extracellular vehicles (EVs). Their research demonstrated that Enterobacteriaceae and the probiotic *Akkermansia muciniphila* (Akk) are significant in enhancing bone formation and suppressing bone metabolism within bone tissues. This is achieved through the release of extracellular vehicles (EVs) into bone tissues, which mitigate bone loss in postmenopausal mice with osteoporosis (51). A recent investigation demonstrated that extracellular vehicles (EVs) released by *Lactobacillus animalis* have the potential to influence the femoral head, mitigating glucocorticoid-induced necrosis through the enhancement of bone formation, vascularization, and anti-apoptotic mechanisms (52).

## 3 The impact of gut microbiota on ageing-related skeletal degenerative diseases

### 3.1 Gut microbiota and osteoporosis

Osteoporosis is a systemic skeletal disorder distinguished by reduced bone density and deterioration of the microarchitecture of bone tissue, resulting in heightened bone fragility and vulnerability to fractures. Osteoporosis is a prevalent disease, and global statistics indicate that the population of individuals with osteoporosis has surpassed 200 million (53). The epidemiological survey conducted on osteoporosis in China revealed that the prevalence of osteoporosis among individuals aged over 50 is 19.2%. Among them, 32.1% are women and 6.9% are men (54). Postmenopausal osteoporosis constitutes the predominant demographic among women affected by osteoporosis.

The dysregulated gut microbiota impacts bone remodeling equilibrium through the mechanisms, resulting in a higher rate of bone resorption compared to bone formation, ultimately causing a reduction in bone mass (54). Numerous inflammatory factors can induce local inflammation and alter the mechanical characteristics of bone, ultimately resulting in osteoporosis. Sjögren et al. discovered that mice with gut microbiota deficiency exhibit approximately 40% higher bone mineral density in the distal femur compared to normal mice. This phenomenon may be attributed to the lower presence of osteoclasts in the gut-deficient mice in comparison to normal mice, while the quantity of osteoblasts does not show significant variance. Consequently, this leads to decreased bone resorption and subsequent bone loss (55). Furthermore, the levels of osteolytic cytokines, such as IL-6 and TNF-α, were notably diminished in the bones of germ-free mice. This suggests that the decrease in osteoclasts in germ-free mice could potentially be attributed to immune system regulation and inflammatory responses in the aging microenvironment. In postmenopausal women with osteoporosis, a decrease in gut microbiota diversity may result in lower circulating estrogen levels. This is because a healthy gut microbiota plays a role in estrogen regulation through the secretion of β-glucuronidase, an enzyme that converts estrogen into its active form. Research has indicated a correlation between estrogen deficiency and osteoporosis (56). Targeting the gut microbiota may exert a therapeutic impact on osteoporosis. Chevalier et al. documented that *Mucinophilic acromyces* have the potential to enhance trabecular volume, junction density, and thickness. This enhancement contributes to the improvement of bone biomechanical strength in adult female and young male mice. Consequently, it aids in the prevention of ovariectomy-induced bone loss and serves as a treatment for osteoporosis (57).

### 3.2 Gut microbiota and fragility fracture repair

As previously stated, the elderly demographic is susceptible to osteoporosis, rendering them more vulnerable to fractures that are harder to mend. An osteoporotic fracture, also known as a fragility fracture, is a type of fracture that occurs due to minor trauma, such as a fall from a standing height or lower (58). It is considered a severe complication of osteoporosis. The most prevalent osteoporotic fracture involves the vertebrae, while the most severe osteoporotic fracture is a hip fracture (59). Osteoporotic fractures pose significant harm and represent a primary contributor to disability and mortality among elderly individuals. Within one year following a hip fracture, approximately 20% of patients may succumb to various complications. Moreover, around 50% of patients may experience disability, leading to a significant decline in their quality of life (60). Additionally, treating fragility fractures in the elderly population is often more challenging. Hence, mending fragility fractures is a research topic of social significance.

The gut microbiota contributes to the process of repairing fragility fractures. Roberts et al. demonstrated that the administration of *Bifidobacterium longum* to elderly mice expedited the formation of bone callus post-fracture, augmented bone healing, and enhanced the biomechanical characteristics of the fractured limb. During the recuperation period following a fracture in mice, the administration of *Bifidobacterium longum* helped preserve the integrity of the intestinal barrier, preventing the fracture-induced decline in gut function and systemic inflammatory responses (61). Liu et al. demonstrated the potential of *probiotic Akkermansia muciniphila* in promoting fracture healing in mice by reducing gut permeability and inflammation (62). As a result, the senescent microenvironment in the new bone is inhibited, contributing to healing at the fracture site.

### 3.3 Gut microbiota and osteoarthritis

Osteoarthritis (OA) is a chronic degenerative disease affecting articular cartilage, commonly observed in middle-aged and elderly individuals. It is characterized by the progressive deterioration of articular cartilage, sclerotic hyperplasia of subchondral bone, the development of periarticular bony outgrowths, and synovial lesions. OA can result in joint pain, dysfunction, and may lead to joint deformity or disability (63).

Lei et al. conducted a fecal sequencing study involving 1,388 subjects, of whom approximately 5% had symptomatic hand osteoarthritis (SHOA). The study revealed notable alterations in pathways associated with amino acid, carbohydrate, and lipid metabolism in the fecal gut of individuals with symptomatic hand osteoarthritis. Furthermore, they discovered a higher relative abundance of Cholera species. *Vibrio desulfuricans species* are present. A decreased relative abundance of *Rousselotiella spp* was observed in the fecal gut (64). In a study, 440 fecal macrogenomes (221 inflammatory arthritis patients and 219 controls) were analyzed. The results suggest an interaction between host genetics, the immune system and gut microbiota in the onset, progression and severity of arthritis (65). Schott et al. discovered a significant reduction in intestinal bifidobacteria in obese rats. This alteration in the gut microbiota composition resulted in the initiation of systemic inflammatory responses, leading to the aggregation of macrophages in the synovial membrane (66). Consequently, this process accelerated the advancement of osteoarthritis. The restoration of gut microbiota through the supplementation of oligofructose resulted in decreased levels of systemic inflammation, potentially mitigating symptoms of arthritis. An elevated population of lactic acid bacteria plays a crucial role in preserving the integrity of the intestinal mucosal barrier, thereby impeding the translocation of bacterial endotoxin or LPS and hindering the progression of osteoarthritis (64). Furthermore, a randomized, double-blind, placebo-controlled clinical study conducted on individuals with knee osteoarthritis demonstrated that the probiotics *Lactobacillus casei Shirota* and *Streptococcus thermophilus* (TCI633) exhibit beneficial effects in ameliorating knee osteoarthritis (67).

### 3.4 Gut microbiota and spinal degeneration disease

Spinal degenerative diseases (SDD) encompass a broad term that includes degenerative conditions affecting spinal structures such as osteoporosis, facet osteoarthritis, intervertebral disk degeneration, lumbar spinal canal stenosis, and spinal sarcopenia (68). Increased inflammatory stress linked to intestinal dysbiosis fosters the senescence of spinal muscle and skeletal cells, leading to the accumulation of diverse senescent cells in spinal structures (18). This accumulation triggers local and systemic inflammation through the senescence-associated secretory phenotype (SASP), thereby playing a role in the progression of SDD.

For a long time it was thought that the intervertebral disc (IVD) was a sterile environment, but now more and more experiments have shown that the IVD also has its own special microbiota and that the microbiota plays an important role in the maintenance of homeostasis in the IVD and in the development of intervertebral disc degeneration (IDD). The gut microbiota has been shown to influence the development of IDD through migration and promotion of inflammation. There are three potential mechanisms widely accepted by which the gut microbiota can induce IDD are: (1) translocation of the bacteria across the gut epithelial barrier and into the intervertebral disc (IVD), (2) regulation of the mucosal and systemic immune system, and (3) regulation of nutrient absorption and metabolites formation at the gut epithelium and its diffusion into the IVD (69). Rajasekaran et al. highlighted that there exists a distinction in the microbiome composition between healthy discs and degenerative or herniated discs. They observed that bacteria residing on discs have the ability to attract additional inflammatory cells through the secretion of inflammatory mediators like IL-6 and TNFα (70), leading to chronic inflammation and thus promoting disc degeneration and the ageing process (71).

Kawaguchi et al. found a correlation between ossification of the posterior longitudinal ligament (OPLL) and leptin as well as chronic inflammation. They also suggested that dysbiosis in gut microbiota influences bone metabolism in connection with chronic inflammation-related diseases and the neurotransmitter leptin. This indicates that gut microbiota dysbiosis may play a role as a pathological factor in OPLL (72). In lumbar spinal stenosis (LSS) patients, inflammatory responses were detected in the typical thickened ligamentum flavum. The factors of lumbar spinal stenosis (LSS), including intervertebral disc disease (IVDD) and facet osteoarthritis (OA), are known to have a relationship with gluteal muscles (GM). Moreover, the correlation between lumbar spinal stenosis (LSS) and chronic inflammation-related disorders like diabetes and metabolic syndrome, along with the inflammation detected in ligament thickening in individuals with LSS, implies a plausible link between LSS and gut microbiota. This suggests the potential existence of a gut-ligament axis in LSS (72).

*In vivo* or *in vitro* experimental studies in humans or animals on the mechanisms and relationships between gut microbiota and the development of degenerative bone diseases are summarized as **Table 1**.

**Table 1:** Summary of studies investigating the impact of gut microbiota on ageing-related skeletal degenerative diseases.

Author	species	Dysbiosis or treatment of microbiota	mechanisms	outcomes
Osteoporosis				
Chevalier et al. (57) Sjögren et al. (55) Wang et al. (54)	Mice Mice Human	Mucinophilic acromyces Deficiency of gut Microbiota Blautia Parabacteroides Lachnoclostridium Klebsiella Ruminococcaceae Prevotella	elevating the polyamine levels increasing osteoclasts and osteolytic cytokines chronic inflammation chronic inflammation chronic inflammation chronic inflammation / /	preventing the ovariectomy-induced bone loss promoting bone mineral density in the distal femur inducing bone loss inducing bone loss inducing bone loss inducing bone loss maintaining bone mass maintaining bone mass
Fragility fracture repair				
Roberts et al. (61) Liu et al. (62)	Mice mice	Bifidobacterium longum Akkermansia muciniphila	preserving the integrity of the intestinal barrier decreasing inflammation	Promoting bone healing Promoting bone healing
osteoarthritis				
Lei et al (67) Schott et al. (66) Weng et al. (64) Lei et al (67) Morimoto T (72)	Human Rat Mice Human Rat	Cholera species Rousselotiella spp bifidobacteria lactic acid bacteria probioticsLactobacillus casei Shirota Streptococcus thermophilus Clostridium butyricum	Increasing inflammation Decreasing inflammation Decreasing inflammation preserve the integrity of the intestinal barrier / / Decreasing inflammation	Promoting OA Demoting OA Demoting OA Demoting OA ameliorating OA ameliorating OA demoting OA
Degeneration disease				
Rajasekaran et al. (70)	Human	Firmicutes Actinobacteria Proteobacteria Bdellovibrio gram-negative bacteria Prevotella	immuno-protection immune-protection immune-protection preying gram-negative bacteria releasing LPS and inducing inflammation increasing inflammation	demoting IDD demoting IDD demoting IDD demoting IDD promoting IDD promoting IDD

## 4 Treatment and prevention of skeletal diseases by means of gut microbiota

Given the increasing recognition of the involvement of the gut microbiota in bone health, various investigations have explored the potential interventions in the gut microbiota for the treatment or prevention of bone diseases by inhibiting the inflammatory response in the senescent microenvironment or directly promoting the osteogenic process.

Probiotic therapy represents a feasible approach. Supplementation with *Lactobacillus animalis* has been documented to offer benefits in averting osteonecrosis of the femoral head via an extracellular vesicular mechanism (52). *Lactobacillus helveticus* HY7801 has demonstrated prophylactic and therapeutic properties in a murine arthritis model by increasing IL-10 expression in CD4+ T cells (73). Moreover, the administration of *Bifidobacterium longum* was found to suppress post-fracture weight reduction and lumbar spine bone density loss in a model of fractures in elderly female mice (61). The oral administration of *Clostridium butyricum* to a rat model with knee osteoarthritis resulted in a notable decrease in serum inflammation and bone metabolism markers, including COX-2 and IL-6 molecules. Additionally, this treatment demonstrated efficacy in maintaining knee cartilage and synovium integrity, as well as reducing fibrous tissue formation (72).

Fecal microbiota transplantation (FMT) involves transferring functional gut amicrobiota from the feces of a healthy individual into the gastrointestinal tract of a patient to restore normal intestinal function. This approach has demonstrated effectiveness in manageing systemic conditions like multiple sclerosis and cancer (74). While there is currently no research on the use of FMT for degenerative bone diseases, this paper highlights the substantial evidence linking intestinal dysbiosis to these conditions. Therefore, FMT has the potential to restore a healthy gut microbiota and may be a promising strategy for treating degenerative bone diseases.

Hormone therapy involving estrogen supplementation, blunting the adverse effects of GCs. Chronic glucocorticoid treatment triggers the senescence of bone-marrow adipocyte (BMAd) lineage cells and induces bone deterioration (75). A study found that delivering recombinant human ANG (rhANG) in vascular endothelial cells to antagonize cellular senescence can blunt the adverse effects of GCs on the growing skeleton (74). Creating a warm environment is also a good strategy. In *in vivo* experiments with mice, a more uniform distribution of gut microbiota abundance was noted in mice exposed to a temperature of 34°C. This led to a decrease in alterations in the transcriptional pathway related to bone remodeling caused by estrogen deficiency, ultimately leading to a notable amelioration of osteoporosis (57). Photobiomodulation (PBM) refers to a light therapy technique that employs non-ionizing light sources such as lasers, light-emitting diodes (LEDs), and broadband light within the visible and infrared spectrum to elicit local or systemic effects. PBM has the potential to modulate the gut microbiota, which plays a crucial role in disease management (76). In a study by Bicknell et al., the impact of infrared laser treatment (904 nm; 700 Hz pulse frequency, 861.3 total joules) administered to the abdomen of breast cancer patients was assessed. The treatment was conducted three times a week for 11 weeks. The study revealed an augmentation in the population of beneficial gut bacteria such as *Akkermansia, Faecalibacterium, and Roseburia*, along with a reduction in pathogenic microorganisms (77).

## 5 Conclusion

This paper examined the fundamental role of the gut microbiota in bone metabolism and the ageing process. It explores the diverse mechanisms through which the gut microbiota influences bone metabolism, consolidates relevant research on the impact of the gut microbiota on conditions such as osteoporosis, fracture healing post-fragility fracture, osteoarthritis, and spinal degenerative diseases. Furthermore, it outlines the connections and current therapeutic strategies for addressing gut microbiota involvement in age-related degenerative skeletal diseases. This paper aims to enhance comprehension of the intestinal-bone axis, improve insight into the pathogenesis of ageing-related bone metabolic disorders, and offer insights for the advancement of therapeutic strategies targeting the gut microbiota. In the realm of therapeutic strategies, the existing approaches predominantly focus on metabolic pathways, exemplified by the use of estrogen supplementation in postmenopausal osteoporosis. Further investigation is warranted to directly target the gut microbiota, potentially influencing the management of bone degenerative conditions. Hormonal therapies have accumulated significant experience; however, further research is needed to address the side effects of estrogenic drugs more effectively. This may involve the exploration of developing estrogen receptor analogs. Warm environment therapy and photobiomodulation offer innovative approaches for addressing degenerative bone diseases. The trend of personalized medicine is increasingly prevalent in healthcare. Sequencing and targeting the individual gut microbiota is a promising approach to fulfill the requirements for personalized medicine. The advancement of additional probiotic medications holds the potential to expand treatment options for degenerative bone conditions. Additionally, fecal transplants represent a promising avenue for personalized therapeutic interventions. The efficacy of Fecal Microbiota Transplantation (FMT) in degenerative bone diseases necessitates further validation through additional experiments. Moreover, careful consideration should be given to the efficacy and safety of FMT therapies. Establishing precise treatment criteria and implementing rigorous screening protocols for donor feces are essential components in this regard.

## Author contributions

LF: Writing – original draft, Writing – review & editing. PZ: Writing – original draft, Writing – review & editing. YW: Writing – original draft, Writing – review & editing. XL: Writing – original draft, Writing – review & editing.

## References

[1] Long F. Building strong bones: molecular regulation of the osteoblast lineage. Nat Rev Mol Cell Biol. (2011) 13:27–38. doi: 10.1038/nrm3254 22189423

[2] Hayflick L. The limited *in vitro* lifetime of human diploid cell strains. Exp Cell Res. (1965) 37:614–36. doi: 10.1016/0014-4827(65)90211-9 14315085

[3] Liu X Wan M. A tale of the good and bad: Cell senescence in bone homeostasis and disease. Int Rev Cell Mol Biol. (2019) 346:97–128. doi: 10.1016/bs.ircmb.2019.03.005 31122396

[4] Lu C Miclau T Hu D Hansen E Tsui K Puttlitz C. Cellular basis for age-related changes in fracture repair. J Orthop Res. (2005) 23:1300–7. doi: 10.1016/j.orthres.2005.04.003.1100230610 PMC2844440 15936915

[5] Li CJ Xiao Y Sun YC He WZ Liu L Huang M. Senescent immune cells release grancalcin to promote skeletal ageing. Cell Metab. (2021) 33:1957–73. doi: 10.1016/j.cmet.2021.08.009 34614408

[6] Chen S Yu Y Xie S Liang D Shi W Chen S. Local H2 release remodels senescence microenvironment for improved repair of injured bone. Nat Commun. (2023) 14:7783. doi: 10.1038/s41467-023-43618-z 38012166 PMC10682449

[7] Ambrosi TH Marecic O McArdle A Sinha R Gulati GS Tong X. Aged skeletal stem cells generate an inflammatory degenerative niche. Nature. (2021) 597:256–62. doi: 10.1038/s41586-021-03795-7 PMC8721524 34381212

[8] Zou NY Liu R Huang M Jiao YR Wei J Jiang Y. Age-related secretion of grancalcin by macrophages induces skeletal stem/progenitor cell senescence during fracture healing. Bone Res. (2024) 12:6. doi: 10.1038/s41413-023-00309-1 38267422 PMC10808101

[9] Cui J Shibata Y Zhu T Zhou J Zhang J. Osteocytes in bone aging: Advances, challenges, and future perspectives. Ageing Res Rev. (2022) 77:101608. doi: 10.1016/j.arr.2022.101608 35283289

[10] Vich Vila A Collij V Sanna S Sinha T Imhann F Bourgonje AR. Impact of commonly used drugs on the composition and metabolic function of the gut microbiota. Nat Commun. (2020) 11:362. doi: 10.1038/s41467-019-14177-z 31953381 PMC6969170

[11] Qin J Li R Raes J Arumugam M Burgdorf KS Manichanh C. A human gut microbial gene catalogue established by metagenomic sequencing. Nature. (2010) 464:59–65. doi: 10.1038/nature08821 20203603 PMC3779803

[12] Luo J Yang H Song BL. Mechanisms and regulation of cholesterol homeostasis. Nat Rev Mol Cell Biol. (2020) 21:225–45. doi: 10.1038/s41580-019-0190-7 31848472

[13] Lian WS Wang FS Chen YS Tsai MH Chao HR Jahr H. Gut microbiota ecosystem governance of host inflammation, mitochondrial respiration and skeletal homeostasis. Biomedicines. (2022) 10:860. doi: 10.3390/biomedicines10040860 35453611 PMC9030723

[14] Cui Y Wang Q Chang R Zhou X Xu C. Intestinal barrier function-non-alcoholic fatty liver disease interactions and possible role of gut microbiota. J Agric Food Chem. (2019) 67:2754–62. doi: 10.1021/acs.jafc.9b00080 30798598

[15] Malesza IJ Malesza M Walkowiak J Mussin N Walkowiak D Aringazina R. High-Fat, western-style diet, systemic inflammation, and gut microbiota: a narrativereview. Cells. (2021) 10:3164. doi: 10.3390/cells10113164 34831387 PMC8619527

[16] Zheng D Liwinski T. Elinav E Interaction between microbiota and immunity in health and disease. Cell Res. (2020) 30:492–506. doi: 10.1038/s41422-020-0332-7 32433595 PMC7264227

[17] Buford TW. (dis)trust your gut: the gut microbiome in age-related inflammation, health, and disease. Microbiome. (2017) 5:80. doi: 10.1186/s40168-017-0296-0 28709450 PMC5512975

[18] Agirman G Yu KB Hsiao EY. Signaling inflammation across the gut-brain axis. Science. (2021) 374:1087–92. doi: 10.1126/science.abi6087 34822299

[19] Smith PM Howitt MR Panikov N Michaud M Gallini CA Bohlooly-Y M. The microbial metabolites, short-chain fatty acids, regulate colonic Treg cell homeostasis. Science. (2013) 341:569–73. doi: 10.1126/science.1241165 PMC3807819 23828891

[20] Jiang M Liu R Liu L Kot A Liu X Xiao W. Identification of osteogenic progenitor cell-targeted peptides that augment bone formation. Nat Commun. (2020) 11:4278. doi: 10.1038/s41467-020-17417-9 32855388 PMC7453024

[21] Luo G Liu H Lu H. Glucagon-like peptide-1(GLP-1) receptor agonists: potential to reduce fracture risk in diabetic patients? Br J Clin Pharmacol. (2016) 81:78–88. doi: 10.1111/bcp.12777 27099876 PMC4693579

[22] Yano JM Yu K Donaldson GP Shastri GG Ann P Ma L. Indigenous bacteria from the gut microbiota regulate host serotonin biosynthesis. Cell. (2015) 161:264–76. doi: 10.1016/j.cell.2015.02.047 PMC4393509 25860609

[23] Cui Y Niziolek PJ MacDonald BT Zylstra CR Alenina N Robinson DR. Lrp5 functions in bone to regulate bone mass. Nat Med. (2011) 17:684–91. doi: 10.1038/nm.2388 PMC3113461 21602802

[24] Stone TW Stoy N Darlington LG. An expanding range of targets for kynurenine metabolites of tryptophan. Trends Pharmacol Sci. (2013) 34:136–43. doi: 10.1016/j.tips.2012.09.006 23123095

[25] Li Z Huang J Wang F Li W Wu X Zhao C. Dual targeting of bile acid receptor-1 (TGR5) and farnesoid X receptor (FXR) prevents estrogen-dependent bone loss in mice. J Bone Miner Res. (2019) 34:765–76. doi: 10.1002/jbmr.3652 30536462

[26] Fukuda S Toh H Hase K Oshima K Nakanishi Y Yoshimura K. Bifidobacteria can protect from enteropathogenic infection through production of acetate. Nature. (2011) 469:543–7. doi: 10.1038/nature09646 21270894

[27] Kim JG Lee E Kim SH. et al, Effects of a Lactobacillus casei 393 fermented milk product on bone metabolism in ovariectomised rats. Int DairyJ. (2009) 19:690–5. doi: 10.1016/j.idairyj.2009.06.009

[28] Wang Y Wang M Chen J Li Y Kuang Z Dende C. The gut microbiota reprograms intestinal lipid metabolism through long noncoding RNA Snhg9. Science. (2023) 381:851–7. doi: 10.1126/science.ade0522 PMC10688608 37616368

[29] Geng J Yu S Zhao H Sun X Li X Wang P. The transcriptional coactivator TAZ regulates reciprocal differentiation of TH17 cells and Treg cells. Nat Immunol. (2017) 18:800–12. doi: 10.1038/ni.3748 28504697

[30] Kong YY Feige U Sarosi I Bolon B Tafuri A Morony S. Activated T cells regulate bone loss and joint destruction in adjuvant arthritis through osteoprotegerin ligand. Nature. (1999) 402:304–9. doi: 10.1038/46303 10580503

[31] Tyagi AM Yu M Darby TM Vaccaro C Li JY Owens JA. The microbial metabolite butyrate stimulates bone formation *via* T regulatory cell-mediatedRegulation of WNT10B expression. Immunity. (2018) 49:1116–1131.e7. doi: 10.1016/j.immuni.2018.10.013 30446387 PMC6345170

[32] Alexander KA Chang MK Maylin ER Kohler T Müller R Wu AC. Osteal macrophages promote in vivo intramembranous bone healing in a mouse tibial injury model J. Bone Miner Res. (2011) 26:1517–32. doi: 10.1002/jbmr.354 21305607

[33] Sun W Meednu N Rosenberg A Rangel-Moreno J Wang V Glanzman J. B cells inhibit bone formation in rheumatoid arthritis by suppressing osteoblast differentiation. Nat Commun. (2018) 9:5127. doi: 10.1038/s41467-018-07626-8 30510188 PMC6277442

[34] Crotti TN Dharmapatni AA Alias E Haynes DR. Osteoimmunology: major and costimulatory pathway expression associated with chronic inflammatory induced bone loss. J Immunol Res. (2015) 2015:281287. doi: 10.1155/2015/281287 26064999 PMC4433696

[35] Dar HY Shukla P Mishra PK Anupam R Mondal RK Tomar GB. Lactobacillus acidophilus inhibits bone loss and increases bone heterogeneity in osteoporotic mice *via* modulating TregTh17 cell balance. Bone Rep. (2018) 8:46–56. doi: 10.1016/j.bonr.2018.02.001 29955622 PMC6019967

[36] Atarashi K Tanoue T Shima T Imaoka A Kuwahara T Momose Y. Induction of colonic regulatory T cells by indigenous Clostridium species. Science. (2011) 331:337–41. doi: 10.1126/science.1198469 PMC3969237 21205640

[37] Ivanov II Atarashi K Manel N Brodie EL Shima T Karaoz U. Induction of intestinal Th17 cells by segmented filamentous bacteria. Cell. (2009) 139:485–98. doi: 10.1016/j.cell.2009.09.033 PMC2796826 19836068

[38] Rooks MG Garrett WS. Gut microbiota, metabolites and host immunity. Nat Rev Immunol. (2016) 16:341–52. doi: 10.1038/nri.2016.42 PMC5541232 27231050

[39] Liao Y Ren Y Luo X Mirando AJ Long JT Leinroth A. Interleukin-6 signaling mediates cartilage degradation and pain in posttraumatic osteoarthritis in a sex-specific manner. Sci Signal. (2022) 15:eabn7082. doi: 10.1126/scisignal.abn7082 35881692 PMC9382892

[40] Tsuji K Maeda T Kawane T Matsunuma A Horiuchi N. Leptin stimulates fibroblast growth factor 23 expression in bone and suppresses renal 1alpha,25-dihydroxyvitamin d3 synthesis in leptin-deficient mice. J Bone Miner Res. (2010) 25:1711–23. doi: 10.1002/jbmr.65 20200981

[41] Yadav VK Oury F Suda N Liu ZW Gao XB Confavreux C. A serotonin-dependent mechanism explains the leptin regulation of bone mass, appetite, and energy expenditure. Cell. (2009) 138:976–89. doi: 10.1016/j.cell.2009.06.051 PMC2768582 19737523

[42] Moon J Koh G. Clinical evidence and mechanisms of high-protein diet-induced weight loss. J Obes Metab Syndr. (2020) 29:166–73. doi: 10.7570/jomes20028 PMC7539343 32699189

[43] Que Q Guo X Zhan L Chen S Zhang Z Ni X. The GLP-1 agonist, liraglutide, ameliorates inflammation through the activation of the PKA/CREB pathway in a rat model of knee osteoarthritis. J Inflammation. (2019) 16:13. doi: 10.1186/s12950-019-0218-y PMC6554939 31182934

[44] Yujiao WANG Qingling JIA Li LI Xiangxiang WANG Jianghong LING. Advances in the relationship between gut microbiota and gastrointestinal motility. World Chin J Digestology. (2021) 29:1020–5.

[45] Kanatani M Sugimoto T Sowa H Kobayashi T Kanzawa M Chihara K. Thyroid hormone stimulates osteoclast differentiation by a mechanism independent of RANKL-RANK interaction. J Cell Physiol. (2004) 201:17–25. doi: 10.1002/jcp.20041 15281085

[46] Zhang J Lu Y Wang Y Ren X Han J. The impact of the intestinal microbiome on bone health. Intractable RareDis Res. (2018) 7:148–55. doi: 10.5582/irdr.2018.01055 PMC6119671 30181933

[47] Ohlsson C Engdahl C Fåk F Andersson A Windahl SH Farman HH. Probiotics protect mice from ovariectomy-induced cortical bone loss. PloS One. (2014) 9:e92368. doi: 10.1371/journal.pone.0092368 24637895 PMC3956931

[48] Yan J Herzog JW Tsang K Brennan CA Bower MA Garrett WS. Gut microbiota induces IGF-1 and promotes bone formation and growth. Proc Natl Acad Sci U.S.A. (2016) 113:E7554–63. doi: 10.1073/pnas.1607235113 PMC5127374 27821775

[49] Zhang L Liu N Shao J Gao D Liu Y Zhao Y. Bidirectional control of parathyroid hormone and bone mass by subfornical organ. Neuron. (2023) 111:1914–1932.e6. doi: 10.1016/j.neuron.2023.03.030 37084721

[50] Liu X Chen C Jiang Y Wan M Jiao B Liao X. Brain-derived extracellular vesicles promote bone-fat imbalance in Alzheimer's disease. Int J Biol Sci. (2023) 19:2409–27. doi: 10.7150/ijbs.79461 PMC10197897 37215980

[51] Liu JH Chen CY Liu ZZ Luo ZW Rao SS Jin L. Extracellular vesicles from child gut microbiota enter into bone to preserve bone mass and strength. Adv Sci (Weinh). (2021) 8:2004831. doi: 10.1002/advs.202004831 33977075 PMC8097336

[52] Chen CY Rao SS Yue T Tan YJ Yin H Chen LJ. Glucocorticoid-induced loss of beneficial gut bacterial extracellular vesicles is associated with the pathogenesis of osteonecrosis. Sci Adv. (2022) 8:eabg8335. doi: 10.1126/sciadv.abg8335 35417243 PMC9007505

[53] Salari N Ghasemi H Mohammadi L Behzadi MH Rabieenia E Shohaimi S. The global prevalence of osteoporosis in the world: a comprehensive systematic review and meta-analysis. J Orthop Surg Res. (2021) 16:609. doi: 10.1186/s13018-021-02772-0 34657598 PMC8522202

[54] Wang L Yu W Yin X Cui L Tang S Jiang N. Prevalence of osteoporosis and fracture in China: the China osteoporosis prevalence study. JAMA Netw Open. (2021) 4:e2121106. doi: 10.1001/jamanetworkopen.2021.21106 34398202 PMC8369359

[55] Ohlsson C Sjögren K. Osteomicrobiology: A new cross-disciplinary research field. Calcif Tissue Int. (2018) 102:426–32. doi: 10.1007/s00223-017-0336-6 PMC5851705 29079994

[56] Qi X Yun C Pang Y Qiao J. The impact of the gut microbiota on the reproductive and metabolic endocrine system. Gut Microbes. (2021) 13:1–21. doi: 10.1080/19490976.2021.1894070 PMC7971312 33722164

[57] Chevalier C Kieser S Çolakoğlu M Hadadi N Brun J Rigo D. Warmth prevents bone loss through the gut microbiota. Cell Metab. (2020) 32:575–590.e7. doi: 10.1016/j.cmet.2020.08.012 32916104 PMC7116155

[58] Kanis JA. Assessment of fracture risk and its application to screening for postmenopausal osteoporosis. Report of a WHO Study Group. Osteoporos Int. (1994) 4(6):368–81. doi: 10.1007/BF01622200 7696835

[59] Zhang J Zhang L Li C Chai W Zhang L Chen H. Clinical guidelines for the diagnosis and treatment of fragility fractures of the pelvis. Orthop Surg. (2023) 15:2195–212. doi: 10.1111/os.13755 PMC10475682 37435891

[60] Loggers S Joosse P Jan Ponsen K. Outcome of pubic rami fractures with or without concomitant involvement of the posterior ring in elderly patients. Eur J Trauma Emerg Surg. (2019) 45:1021–9. doi: 10.1007/s00068-018-0971-2 29947849

[61] Roberts JL Golloshi M Harding DB Conduah M Liu G Drissi H. Bifidobacterium longum supplementation improves age-related delays in fracture repair. Ageing Cell. (2023) 22:e13786. doi: 10.1111/acel.13786 PMC10086533 36704918

[62] Liu JH Yue T Luo ZW Cao J Yan ZQ Jin L. Akkermansia muciniphila promotes type H vessel formation and bone fracture healing by reducing gut permeability and inflammation. Dis Model Mech. (2020) 13(11):dmm043620. doi: 10.1242/dmm.043620 33033107 PMC7725610

[63] Lin J Fransen M Kang X Li H Ke Y Wang Z. Marked disability and high use of nonsteroidal antiinflammatory drugs associated with knee osteoarthritis in rural China: a cross-sectional population-based survey. Arthritis Res Ther. (2010) 12:R225. doi: 10.1186/ar3212 21190567 PMC3046538

[64] Weng Q Chen Q Jiang T Zhang Y Zhang W Doherty M. Global burden of early-onset osteoarthritis, 1990-2019: results from the Global Burden of Disease Study 2019. Ann Rheum Dis. (2024) 83:915–25. doi: 10.1136/ard-2023-225324 38429104

[65] Thompson KN Bonham KS Ilott NE Britton GJ Colmenero P Bullers SJ. Alterations in the gut microbiome implicate key taxa and metabolic pathways across inflammatory arthritis phenotypes. Sci Transl Med. (2023) 15:eabn4722. doi: 10.1126/scitranslmed.abn4722 37494472

[66] Schott EM Farnsworth CW Grier A Lillis JA Soniwala S Dadourian GH. Targeting the gut microbiome to treat the osteoarthritis of obesity. JCI Insight. (2018) 3:e95997. doi: 10.1172/jci.insight.95997 29669931 PMC5931133

[67] Lei M Guo C Wang D Zhang C Hua L. The effect of probiotic Lactobacillus casei Shirota on knee osteoarthritis: a randomised double-blind, placebo-controlled clinical trial. Benef Microbes. (2017) 8:697–703. doi: 10.3920/BM2016.0207 28726510

[68] Masuda K Aota Y Muehleman C Imai Y Okuma M Thonar EJ. A novel rabbit model of mild, reproducible disc degeneration by an annulus needle puncture: correlation between the degree of disc injury and radiological and histological appearances of disc degeneration. Spine (Phila Pa 1976). (2005) 30:5–14. doi: 10.1097/01.brs.0000148152.04401.20 15626974

[69] Li W Lai K Chopra N Zheng Z Das A Diwan AD. Gut-disc axis: A cause of intervertebral disc degeneration and low back pain? Eur Spine J. (2022) 31:917–25. doi: 10.1007/s00586-022-07152-8 35286474

[70] Lisiewski LE Jacobsen HE Viola DCM Kenawy HM Kiridly DN Chahine NO. Intradiscal inflammatory stimulation induces spinal pain behavior and intervertebral disc degeneration in *vivo* . FASEB J. (2022) 38(1):e23364. doi: 10.1096/fj.202300227R PMC10795732 38091247

[71] Li Y Luo W Deng Z Lei G. Diet-gut microbiota axis in osteoarthritis: a possible role. Mediat Inflammation. (2016) 2016:3495173. doi: 10.1155/2016/3495173 PMC5005536 27610004

[72] Morimoto T Kobayashi T Kakiuchi T Esaki M Tsukamoto M Yoshihara T. Gut-spine axis: a possible correlation between gut microbiota and spinal degenerative diseases. Front Microbiol. (2023) 14:1290858. doi: 10.3389/fmicb.2023.1290858 37965563 PMC10641865

[73] Kim JE Chae CS Kim GC Hwang W Hwang JS Hwang SM. Lactobacillus helveticus, suppresses experimental rheumatoid arthritis by reducing inflammatory T cell responses. J Funct Foods. (2015) 13:350–62. doi: 10.1016/j.jff.2015.01.002

[74] Liu X Chai Y Liu G Su W Guo Q Lv X. Osteoclasts protect bone blood vessels against senescence through the angiogenin/plexin-B2 axis. Nat Commun. (2021) 12:1832. doi: 10.1038/s41467-021-22131-1 33758201 PMC7987975

[75] Liu X Gu Y Kumar S Amin S Guo Q Wang J. Oxylipin-PPARγ-initiated adipocyte senescence propagates secondary senescence in the bone marrow. Cell Metab. (2023) 35:667–684.e6. doi: 10.1016/j.cmet.2023.03.005 37019080 PMC10127143

[76] Montazeri K Farhadi M Fekrazad R Chaibakhsh S Mahmoudian S. Photobiomodulation therapy in mood disorders: a systematic review. Lasers Med Sci. (2022) 37:3343–51. doi: 10.1007/s10103-022-03641-w 36404359

[77] Bicknell B Laakso EL Liebert A Kiat H. Modifying the microbiome as a potential mechanism of photobiomodulation: A case report. Photobiomodul Photomed Laser Surg. (2022) 40:88–97. doi: 10.1089/photob.2021.0057 34962422

